# P-990. Impact of a Pharmacy Resident-led Antiretroviral Stewardship Program (ARVSP)

**DOI:** 10.1093/ofid/ofaf695.1189

**Published:** 2026-01-11

**Authors:** Kyle W Furlow, Madeline Cooper, Natalie Delozier, Sahand Golpayegany, Sarah M Rowe, Benjamin Albrecht, Sarah B Green

**Affiliations:** Emory Johns Creek Hospital, Atlanta, Georgia; University of Alabama Birmingham Medicine, Birmingham, Alabama; The Christ Hospital, Cincinnati, Ohio; Wellstar Cobb Medical Center, Marietta, GA; Emory Long Term Acute Care Hospital, Decatur, Georgia; Emory University Hospital, Atlanta, Georgia; Emory University Hospital, Atlanta, Georgia

## Abstract

**Background:**

Due to a 120% increase in patients living with HIV (PLWH) admitted to our institution over the prior 3-year period, a pharmacy resident-driven ARVSP was implemented to augment the existing antimicrobial stewardship program. This study evaluated the feasibility and impact of an ARVSP conducted by non-infectious diseases trained, post-graduate year 1 (PGY1) pharmacy residents at a large, academic medical center.

**Figure 1:**
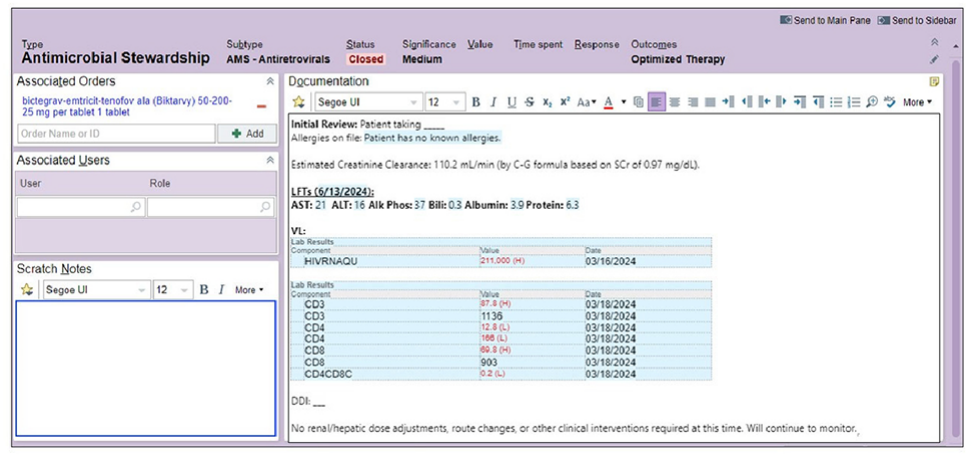
Chart review documentation via an Epic i-Vent.

**Table 1:**
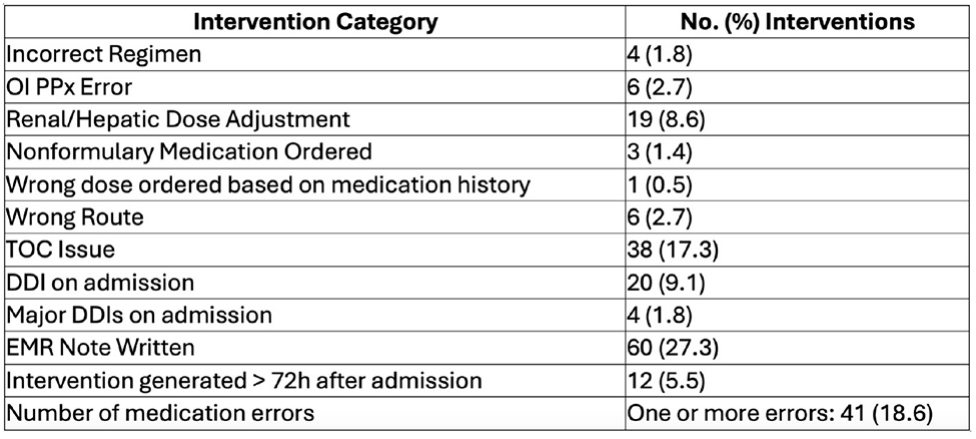
Summary Data. OI = Opportunistic Infections, PPx = Prophylaxis, TOC = Transitions of Care, DDI = Drug-Drug Interaction, EMR = Electronic Medical Record

**Methods:**

This was a single-center, retrospective pilot study. From 7/1/2022 to 2/1/2023 the PGY1 ARVSP followed a standard operating procedure (SOP) for evaluating medication regimens of admitted PLWH on antiretroviral (ARV) therapy. The SOP included a checklist to minimize prescribing errors, drug-drug interactions (DDI), and optimize ARV regimens for admitted patients, particularly with changing clinical status. Daily chart reviews included external prescription history, outpatient notes, inpatient medications, and clinical status (Figure 1). Patient interviews were conducted to confirm ARV regimens, drug allergies, home medications, outpatient HIV follow-up plan, and barriers to medication access.

**Table 2:**
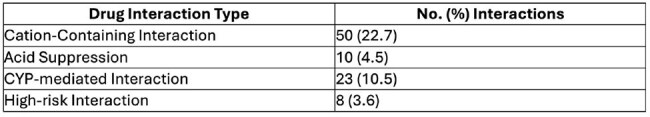
Drug Interaction Summary. CYP = cytochrome P450, High-risk = anticoagulants, antiplatelets, antiepileptics, antiarrhythmics, HMG-CoA reductase inhibitors, antipsychotics, and antibiotics

**Table 3:**
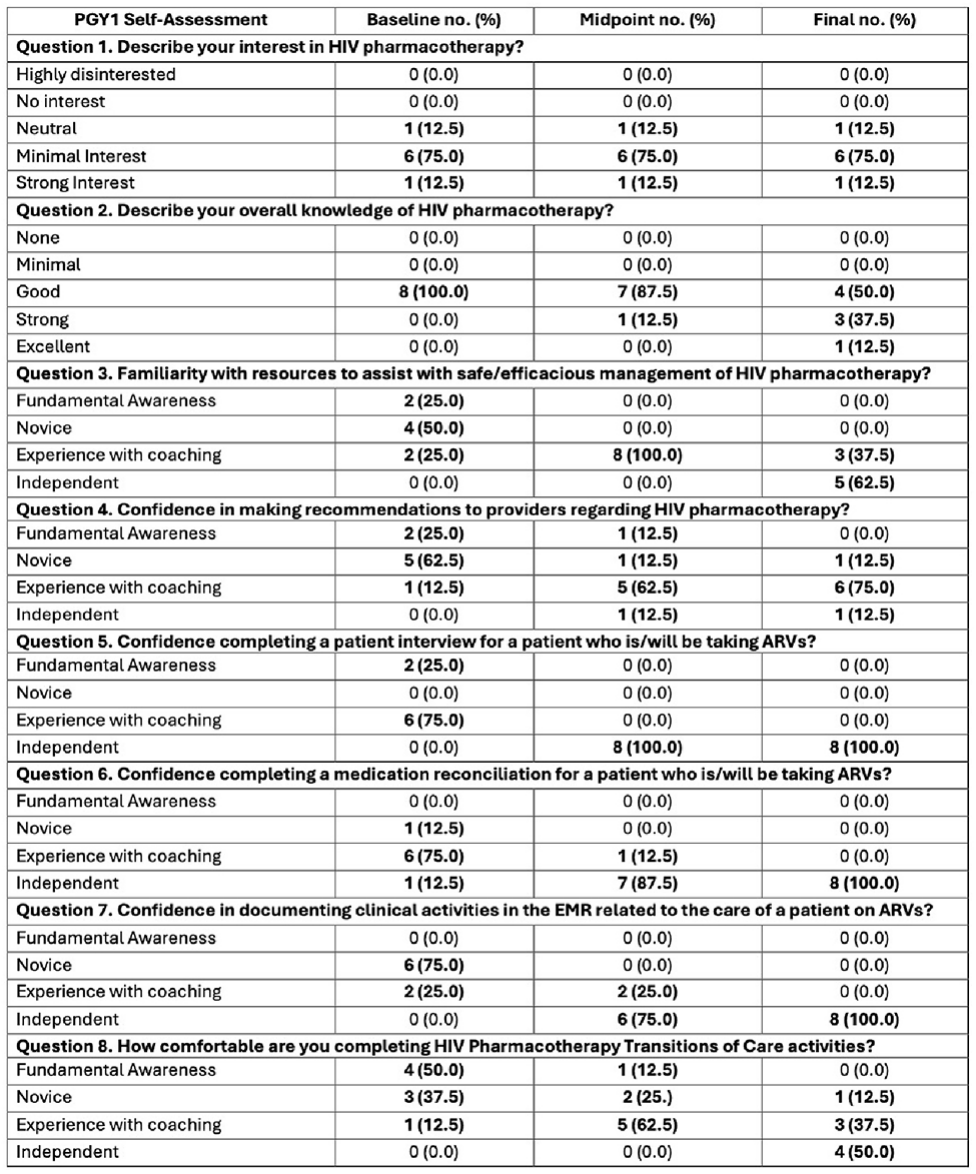
PGY1 individual survey responses. PGY1 = post graduate year 1; HIV = human immunodeficiency virus; ARVs = antiretrovirals

**Results:**

A total of 220 patients were reviewed during the 7-month ARVSP pilot period. Over 100 unique interventions were identified by the ARVSP team, including DDI, dosage adjustments, need for opportunistic infection prophylaxis, incorrect route of administration, or incorrect dose/regimen resumed inpatient (Table 1). The majority of the DDI identified involved potential issues with drug absorption but a significant number of high-risk and CYP-mediated DDI were also found (Table 2). PGY1 residents were surveyed throughout the pilot and self-identified improvement to the level of independence in patient interviewing, reconciling medications, and documenting clinical activities in the medical record (Table 3).

**Conclusion:**

PGY1 residents implemented ARVSP activities into their daily workflow without limiting their direct patient care responsibilities. They successfully identified ARV medication errors and reported improvements in their ability to manage ARV pharmacotherapy. PGY1 pharmacy residency programs can adapt similar ARVSPs to augment their training programs and support medication optimization in this high-risk patient population.

**Disclosures:**

All Authors: No reported disclosures

